# Age and sex specific effects of *APOE* genotypes on ischemic heart disease and its risk factors in the UK Biobank

**DOI:** 10.1038/s41598-021-88256-x

**Published:** 2021-04-29

**Authors:** Mengyu Li, Jie V. Zhao, Man Ki Kwok, C. Mary Schooling

**Affiliations:** 1 School of Public Health, Li Ka Shing, Faculty of Medicine, The University of Hong Kong, 1/F, Patrick Manson Building (North Wing), 7 Sassoon Road, Pokfulam, Hong Kong China; 2 Graduate School of Public Health and Health Policy, City University of New York, New York, NY 10017 USA

**Keywords:** Clinical genetics, Haplotypes, Cardiology

## Abstract

*APOE* genotypes are associated with ischemic heart disease (IHD), several other cardiovascular diseases and dementia. Previous studies have not comprehensively considered all genotypes, especially ε2ε2, nor associations by age and sex, although IHD incidence differs by sex. In the UK Biobank, including 391,992 white British participants, we compared effects of *APOE* genotypes on IHD and its risk factors. Compared to the ε3ε3 genotype, ε2ε2 was not clearly associated with IHD but was associated with lower plasma apolipoprotein B (apoB). The ε2ε3 genotype conferred lower IHD risk, systolic blood pressure (SBP), pulse pressure and plasma apoB than ε3ε3. ε3ε4 and ε4ε4 conferred higher IHD risk, higher pulse pressure and plasma apoB, but lower glycated haemoglobin (HbA1c) than ε3ε3. The associations by age and sex were fairly similar, except ε2ε2 compared to ε3ε3 was marginally positively associated with IHD in the younger age group and nominally inversely associated with SBP in men. ε3ε4 compared to ε3ε3 was nominally positively associated with SBP in women. *APOE* genotypes affect IHD risk increasingly from ε2ε3, ε3ε3, ε3ε4 to ε4ε4, with similar patterns for pulse pressure and plasma apoB, but not for diabetes. Associations with blood pressure differed by sex. Greater understanding of products of *APOE* and their effects might generate targets of intervention.

## Introduction

Ischemic heart disease (IHD) is the leading cause of mortality and morbidity globally^1^, with associated costs and economic impact^2^. Diabetes is an important co-morbidity with a heavy disease burden^1^. The development of cardiovascular disease (CVD) is complex, with genetic background a contributing factor^3^. So far, several genes, such as *LDLR*, *PCSK9*, *HMGCR* and *SLC12A1* have been recognized for their role in IHD, and correspondingly are therapeutic targets^4^. Identifying genetic variants that strongly affect CVD can help elucidate targets for intervention^5,6^. Notably, sex differences in the incidence of IHD are greater than the differences for diabetes^7^, raising the possibility of sex-specific targets. The importance of sex-specific investigations is increasingly recognized as a means of investigating disparities and finding more target interventions^8^. Although prevention and treatment of CVD have improved immensely, declines in CVD mortality have stalled in the United States since 2011^9^, indicating new prevention strategies are needed^10^, but have become increasingly difficult to identify^11^. In this situation, re-assessment of “backgrounded” or overlooked targets, such as apolipoprotein E (apoE)^12^, is increasingly being undertaken^13,14^.

The apoE protein has three isoforms (apoE2, apoE3 and apoE4) coded by three allelic variants, known as ε2, ε3 and ε4, which form six common genotypes (ε2ε2, ε2ε3, ε2ε4, ε3ε3, ε3ε4 and ε4ε4)^15,16^, determined by two single-nucleotide polymorphisms (SNPs), i.e., rs429358 and rs7412^16^. *APOE* genetic variants are a well-known genetic determinant of longevity^17^, affecting androgens^18^ whose relevance to human health in terms of the evolutionary biology trade-off between reproduction and longevity, is increasingly recognized^19^. ε3 (rs429358-T, rs7412-C) carriage is most common with a frequency of around 78.3%^20^, although whether ε3 or ε4 is the ancestral form is controversial^21,22^. ε2 (rs429358-T, rs7412-T) carriers compared to ε3ε3 carriers have lower risk of IHD^16,23–26^, lower risk of hypertension^27^, higher risk of type 2 diabetes^28^, and lower plasma apolipoprotein B (apoB)^29–32^. A recent phenome-wide association study (PheWAS) showed ε2 associated with a wide range of age-related outcomes^13^, while another suggested the ε2ε2 genotype was positively associated with peripheral vascular disease and thromboembolism^14^. Conversely, ε4 (rs429358-C, rs7412-C) carriers compared to ε3ε3 have a higher risk of IHD^16,23–26,33^, hypertension^27^, and higher plasma apoB^29–32^ with both positive and inverse associations with type 2 diabetes observed^14,28^.

Most studies of ε2 carriage are based on the ε2ε3 genotype, while effects of ε2ε2 are less well-established, due to the low frequency of ε2. *APOE* is known to be a key determinant of longevity^17^ and aging^13^. However, some of the evidence for effects on CVD concerns older people^34,35^. Observational studies in older people can be difficult to interpret because those who have died before recruitment from the exposure, from the outcome or from a competing risk of the outcome are inevitably excluded from the study by prior death, meaning the full effect on the outcome cannot be observed due to selection bias. The small magnitude of genetic associations means genetic studies may be more vulnerable to such bias than traditional observational studies. Studies in younger people are less open to such selection bias^36^, drawing attention to the importance of age-specific associations which has not been considered in previous studies^13,14^. *APOE* genetic variants may affect androgens^18^, and androgens affect IHD, suggesting the associations could differ by sex, but previous PheWAS did not consider sex-specific associations^13,14^. Nowadays, the importance of investigating sex-specific associations has been recognized and encouraged to provide insights hopefully to reduce disparities and better target interventions^8^. Here, we made use of a large population-based study of people of mean age 56.9 years, i.e., the UK Biobank, with high-quality indicators of potential population stratification, to assess effects of common *APOE* genotypes on IHD overall, stratified by age at recruitment to obtain estimates less open to selection bias, and by sex to provide insights about differences by sex. We also similarly assessed associations with traditional IHD risk factors, i.e., blood pressure, type 2 diabetes, glycated hemoglobin (HbA1c), and with plasma apoB given it is increasingly being considered as an important cause of IHD^37–39^. We also included low-density lipoprotein (LDL) cholesterol as a positive control outcome, given *APOE* genotypes are known to affect LDL cholesterol^23^. Specifically, LDL cholesterol is lower in ε2ε2, ε2ε3 and ε2ε4 carriers, but higher in ε3ε4 and ε4ε4 carriers, than in ε3ε3 carriers^23^.

## Methods

### Data sources

The UK Biobank is one of the largest ongoing cohort studies worldwide, which recruited more than 500,000 participants in 2006–2010 intended to be aged 40–69 years from the UK (specifically Great Britain)^40^. At baseline comprehensive assessments were made, and samples collected. Follow-up via record linkage to hospitalizations and death registration is ongoing^40^. Genotyping was based on two highly similar genotyping arrays (95% of marker content shared), i.e., the Applied Biosystems UK BiLEVE Axiom Array (50,000 participants) and the Applied Biosystems UK Biobank Axiom Array (450,000 participants), and was imputed using the Haplotype Reference Consortium (HRC) and the UK10K haplotype resource^41^. To avoid confounding by population stratification, only participants of white British ancestry were included here. For quality control, we also excluded participants with genetic and reported sex mismatch, sex chromosome aneuploidy, genotyping missing rate > 1.5%, or extensive relatedness (more than 10 putative third degree relatives).

### Exposures

We compared the *APOE* genotypes (ε2ε2, ε2ε3, ε2ε4, ε3ε4 and ε4ε4) with the *APOE* ε3ε3 genotype. Genotypes were based on combinations of haplotypes derived from rs429358 and rs7412 as shown in Supplementary Table 1.

### Outcomes

The primary outcome was IHD, and the secondary outcomes were systolic blood pressure (SBP) (mmHg), diastolic blood pressure (DBP) (mmHg), pulse pressure (mmHg), type 2 diabetes with or without complications, HbA1c (mmol/mol) and plasma apoB (g/L), with LDL cholesterol (mmol/L) as a positive control outcome. Disease outcomes were based on self-report at baseline, and subsequent record linkage to both primary and secondary diagnoses of hospital episodes, and both primary and secondary causes of death (Supplementary Table 2), using individual level data updated to August 2019. SBP and DBP were from the average of two measurements (automated readings using Omron) made during the initial assessment, with 15 mmHg and 10 mmHg added to SBP and DBP respectively for people on anti-hypertensive medication^42^. Taking medication use into account in this fashion may not reflect the effectiveness of medication use, so blood pressure without adjustment for medication use was used in sensitivity analyses. Pulse pressure was the difference between SBP and DBP. HbA1c was measured using HPLC analysis (Bio-Rad VARIANT II Turbo). Plasma apoB was measured using immunoturbidimetric analysis (Beckman Coulter AU5800). LDL cholesterol was measured using enzymatic protective selection analysis (Beckman Coulter AU5800), with 1.1 mmol/L added to LDL cholesterol for people on cholesterol lowering medication^43^. Similarly, LDL cholesterol without adjustment for medication use was used in sensitivity analyses.

### Potential confounders

The first 40 principal components provided by the UK Biobank were used to control for population stratification. These had previously been derived using an algorithm (fastPCA), based on 407,219 unrelated, high quality samples and 147,604 high quality markers, aiming to capture population structure at both sample and marker level. The principal components are associated with self-reported ethnic background and population structure at sub-continental geographic scales^41^.

### Statistical analysis

We used χ^2^ tests or ANOVA to assess the associations of *APOE* genotype with baseline characteristics, including age, sex, body mass index (BMI), smoking status, alcohol use, indicators of socio-economic position, including education, average total household income before tax and Townsend deprivation index, physical activity based on the International Physical Activity Questionnaires (IPAQ), and use of medication for lowering cholesterol, blood pressure or for diabetes, and use of exogenous hormones, including hormone replacement therapy and oral contraceptives by women.

Genetic associations obtained using logistic or linear regression were adjusted for age at recruitment, square of age at recruitment, sex, sex × age at recruitment, sex × square of age at recruitment, genotyping array and the first 40 principal components, as previously^44^. We also stratified the analyses by age at recruitment using a traditional cut-off of 60 years^45^ and by sex, with similar adjustments, and compared differences using a z-test^46^. Given in this study we tested associations of *APOE* genotypes with seven outcomes including one main outcome IHD and six secondary, possibly correlated, outcomes, we used a Bonferroni correction to account for multiple hypotheses testing, giving a *p* value threshold of 0.007 (0.05/7).

All statistical analyses were conducted using R version 3.6.2. This research has been conducted using the UK Biobank Resource under Application number (42468). The UK Biobank has already received ethical approval from the North West Multi-centre Research Ethics Committee (MREC) which covers the UK. It also got the approval from the Patient Information Advisory Group (PIAG) in England and Wales, and from the Community Health Index Advisory Group (CHIAG) in Scotland. The study protocol conforms to the ethical guidelines of the 1975 Declaration of Helsinki.

### Ethics approval and consent to participate

This research has been conducted using the UK Biobank Resource under Application number (42468). The UK Biobank has already received ethical approval from the Research Ethics Committee and participants provided written informed consent. The study protocol conforms to the ethical guidelines of the 1975 Declaration of Helsinki.

### Consent for publication

Not applicable.

## Results

After selecting on white British ancestry and quality control criteria, 391,992 participants remained. Among these participants, 33,490 had IHD, and 18,211 had type 2 diabetes.

**Figure 1:**
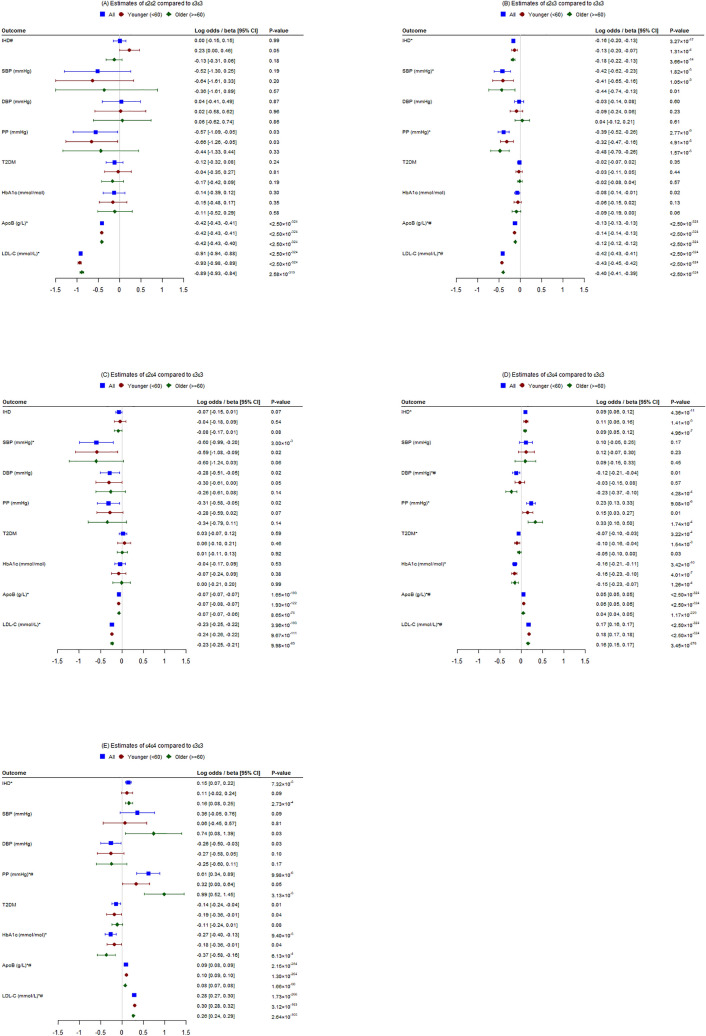
Associations of common *APOE* genotypes (ε2ε2, ε2ε3, ε2ε4, ε3ε4 and ε4ε4) with ischemic heart disease (IHD) and its risk factors [systolic blood pressure (SBP), diastolic blood pressure (DBP), pulse pressure (PP), type 2 diabetes (T2DM), glycated hemoglobin (HbA1c), plasma apolipoprotein B (apoB), and low-density lipoprotein cholesterol (LDL-C)] compared to ε3ε3 genotype overall and by age group in the UK Biobank (*P value < 0.007, ^#^*P* value from z tests comparing differences by age group < 0.05).

**Figure 2:**
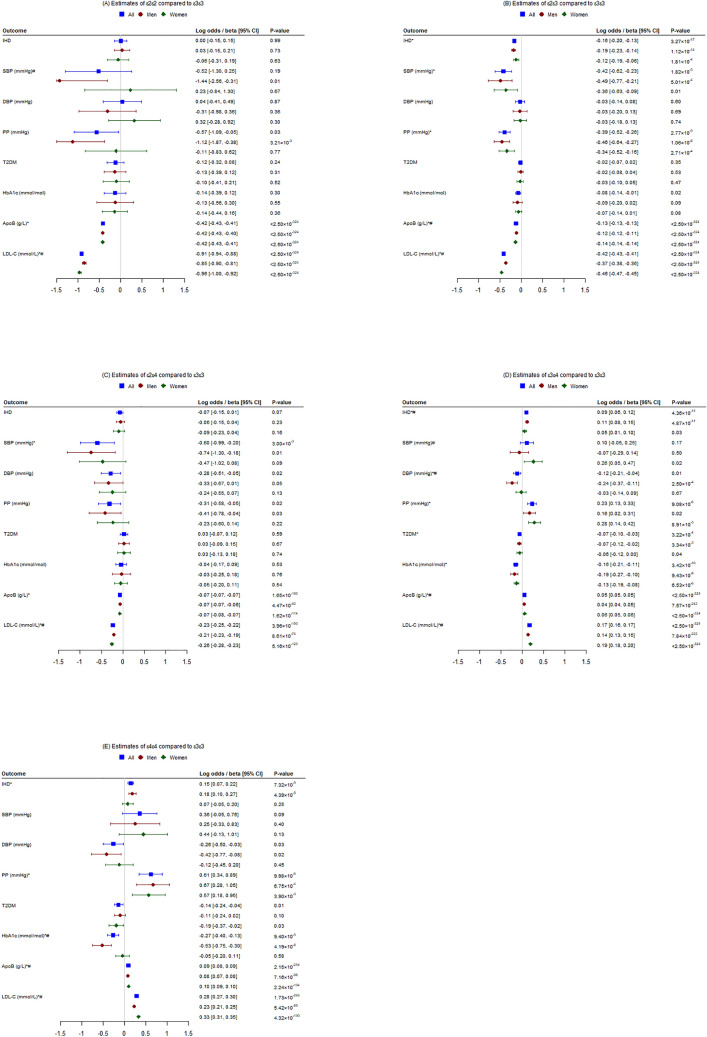
Associations of common *APOE* genotypes (ε2ε2, ε2ε3, ε2ε4, ε3ε4 and ε4ε4) with ischemic heart disease (IHD) and its risk factors [systolic blood pressure (SBP), diastolic blood pressure (DBP), pulse pressure (PP), type 2 diabetes (T2DM), glycated hemoglobin (HbA1c), plasma apolipoprotein B (apoB), and low-density lipoprotein cholesterol (LDL-C)] compared to ε3ε3 genotype overall and by sex in the UK Biobank (*P value < 0.007, ^#^*P* value from z tests comparing differences by sex < 0.05).

Table 1 shows baseline characteristics of the 391,992 participants overall and by *APOE* genotype. Mean age was 56.9 years, and 54.1% were women. Mean BMI was 27.4 kg/m^2^. Just over half had never smoked, 10% were current and 35% were previous smokers. More than 90% were current alcohol users. No differences by *APOE* genotype were evident for sex, smoking, alcohol use, socio-economic position, use of insulin or hormones (in women). Participants with the ε2ε2 or ε4ε4 genotype were slightly younger than the others. ε4ε4 carriers had lower BMI and were more physically active than others. ε3ε4 and ε4ε4 carriers were more likely to take cholesterol or blood pressure lowering medication than others. As expected ε2ε2, ε2ε3 and ε2ε4 carriers had lower LDL cholesterol than ε3ε3, and ε3ε4 and ε4ε4 had higher LDL cholesterol than ε3ε3 (Figs. 1 and 2).

**Table 1:** Characteristics of the participants by common *APOE* genotypes in the UK Biobank at baseline.

*APOE* genotypes	Overall	ε2ε2	ε2ε3	ε2ε4	ε3ε3	ε3ε4	ε4ε4	*P* value
No. participants	391,992	2534	48,551	10,058	227,845	93,560	9444	–
Age, y, mean (SD)	56.91 (8.00)	56.70 (8.10)	56.98 (7.98)	56.79 (8.02)	56.94 (8.00)	56.83 (7.99)	56.74 (7.99)	1.39 × 10^–4^
**Sex (%)**								0.75
Women	54.10	55.25	54.05	53.76	54.15	54.05	53.75	
Men	45.90	44.75	45.95	46.24	45.85	45.95	46.25	
BMI, kg/m^2^, mean (SD)	27.41 (4.76)	27.68 (5.05)	27.51 (4.79)	27.39 (4.75)	27.43 (4.75)	27.36 (4.76)	27.10 (4.66)	3.31 × 10^–16^
**Smoking status (%)**								0.37
Never	54.40	55.49	54.32	55.36	54.38	54.40	54.11	
Previous	35.14	34.25	34.94	34.49	35.12	35.32	35.66	
Current	10.11	9.91	10.40	9.78	10.15	9.93	9.86	
Prefer not to answer	0.35	0.36	0.33	0.37	0.35	0.34	0.37	
**Alcohol drinker status (%)**								0.52
Never	3.14	3.04	3.32	3.28	3.12	3.07	3.13	
Previous	3.43	3.43	3.39	3.71	3.44	3.38	3.64	
Current	93.35	93.37	93.20	92.93	93.36	93.47	93.15	
Prefer not to answer	0.09	0.16	0.09	0.08	0.09	0.08	0.07	
**Education (%)**								0.44
College or University degree	30.65	30.98	30.24	30.52	30.62	30.84	31.51	
A levels/AS levels or equivalent	11.18	10.50	11.14	10.80	11.21	11.18	11.53	
O levels/GCSEs or equivalent	22.15	22.53	22.13	22.51	22.11	22.25	21.81	
CSEs or equivalent	5.53	5.92	5.52	5.84	5.51	5.52	5.62	
NVQ or HND or HNC or equivalent	6.72	6.55	6.77	6.29	6.77	6.65	6.37	
Other professional qualifications eg: nursing, teaching	5.13	4.66	5.26	5.26	5.13	5.05	5.09	
None of the above	17.71	18.00	18.01	17.85	17.71	17.59	16.97	
Prefer not to answer	0.84	0.71	0.86	0.83	0.84	0.84	1.02	
Missing	0.09	0.16	0.07	0.11	0.10	0.09	0.06	
**Average total household income before tax (%)**								0.06
Less than £18,000	19.29	18.75	19.33	19.04	19.31	19.23	19.39	
£18,000 to 30,999	22.22	21.63	22.17	21.55	22.30	22.17	22.12	
£31,000 to 51,999	22.68	23.52	22.29	23.04	22.70	22.72	23.33	
£52,000 to 100,000	17.40	17.05	17.60	17.65	17.29	17.55	17.28	
Greater than £100,000	4.44	4.03	4.35	4.27	4.51	4.40	4.13	
Prefer not to answer	9.71	10.06	9.92	10.03	9.72	9.55	9.39	
Do not know	3.93	4.70	4.01	4.12	3.84	4.04	4.10	
Missing	0.33	0.28	0.32	0.31	0.33	0.35	0.26	
Townsend deprivation index^a^, mean (SD)	− 1.56 (2.93)	− 1.57 (2.93)	-1.57 (2.93)	− 1.59 (2.90)	− 1.55 (2.93)	− 1.56 (2.94)	− 1.59 (2.93)	0.57
**IPAQ activity group**^**b**^								1.19 × 10^–5^
Low	15.08	15.04	15.27	15.35	15.20	14.76	14.09	
Moderate	33.03	32.00	33.39	32.77	33.06	32.82	32.85	
High	32.81	32.24	32.09	32.96	32.70	33.34	34.06	
Missing	19.08	20.72	19.25	18.92	19.04	19.08	19.00	
**Cholesterol lowering medication**								5.22 × 10^–273^
Yes	17.48	15.67	12.88	15.31	17.34	20.00	22.30	
No	81.93	83.70	86.50	84.13	82.06	79.43	77.13	
Prefer not to answer	0.05	0.12	0.04	0.06	0.05	0.04	0.05	
Do not know	0.55	0.51	0.58	0.50	0.55	0.53	0.52	
**Blood pressure medication**								7.19 × 10^–4^
Yes	20.90	20.21	20.07	19.95	21.08	21.00	21.27	
No	78.50	79.16	79.31	79.49	78.32	78.43	78.16	
Prefer not to answer	0.05	0.12	0.04	0.06	0.05	0.04	0.05	
Do not know	0.55	0.51	0.58	0.50	0.55	0.53	0.52	
**Insulin**								0.83
Yes	1.04	0.95	1.02	1.04	1.07	1.00	1.03	
No	98.36	98.42	98.36	98.40	98.33	98.42	98.40	
Prefer not to answer	0.05	0.12	0.04	0.06	0.05	0.04	0.05	
Do not know	0.55	0.51	0.58	0.50	0.55	0.53	0.52	
**Hormone replacement therapy/Oral contraceptive pill or minipill (women only)**								0.61
Yes	9.76	8.57	9.61	10.04	9.71	9.94	9.97	
No	89.85	90.93	89.97	89.55	89.90	89.68	89.74	
Prefer not to answer	0.04	0.14	0.04	0.07	0.04	0.04	0.02	
Do not know	0.35	0.36	0.37	0.33	0.35	0.34	0.28	

^a^Townsend deprivation index for each participant was calculated based on the preceding national census output areas in which their postcode is located.

^b^A high activity group was defined as levels of physical activity equates to approximately at least one hour per day or more, of at least moderate-intensity activity above the basal level of physical activity. A moderate activity group was defined as levels of physical activity equates to half an hour of at least moderate-intensity physical activities on most days. A low activity group was defined as levels of physical activity not meeting any of the criteria for either of the high or moderate activity group.

### Associations of ε2ε2 genotype with IHD and its risk factors

ε2ε2 carriers differed from ε3ε3 in having nominally lower pulse pressure and lower plasma apoB but otherwise had similar risk of IHD, similar SBP and DBP, and similar risk of type 2 diabetes and similar HbA1c (Figs. 1A and 2A). ε2ε2 was marginally positively associated with IHD in younger people, which differed from the estimate in older people (Fig. 1A, Supplementary Table 3). ε2ε2 was nominally inversely associated with SBP in men, which differed from the estimate in women (Fig. 2A, Supplementary Table 4). Results for blood pressure without adjustment for medication use were similar (Supplementary Table 5).

### Associations of ε2ε3 genotype with IHD and its risk factors

ε2ε3 carriers differed from ε3ε3 in having lower risk of IHD, lower SBP, pulse pressure and plasma apoB, and nominally lower HbA1c, but similar DBP and risk of diabetes (Figs. 1B and 2B). Associations were generally similar by age and sex, except that magnitude of estimates for plasma apoB were significantly larger in younger people (Fig. 1B, Supplementary Table 3) and women (Fig. 2B, Supplementary Table 4). Results for blood pressure without adjustment for medication use were similar (Supplementary Table 6).

### Associations of ε2ε4 genotype with IHD and its risk factors

ε2ε4 carriers differed from ε3ε3 carriers in having lower SBP and plasma apoB, and nominally lower DBP, pulse pressure, but similar IHD and type 2 diabetes risk and HbA1c (Figs. 1C and 2C). Associations were similar by age (Fig. 1C, Supplementary Table 3) and sex (Fig. 2C, Supplementary Table 4). Results for blood pressure without adjustment for medication use were similar (Supplementary Table 7).

### Associations of ε3ε4 genotype with IHD and its risk factors

ε3ε4 carriers differed from ε3ε3 carriers in having higher risk of IHD, pulse pressure and plasma apoB, but lower DBP, type 2 diabetes risk and HbA1c. (Figs. 1D and 2D). Estimates for DBP and plasma apoB were significantly different by age (Fig. 1D, Supplementary Table 3) and those for SBP, DBP and plasma apoB were significantly different by sex (Fig. 2D, Supplementary Table 4). Results for blood pressure without adjustment for medication use were similar (Supplementary Table 8).

### Associations of ε4ε4 genotype with IHD and its risk factors

ε4ε4 carriers differed from ε3ε3 carriers in having higher risk of IHD, pulse pressure and plasma apoB, while having lower HbA1c, but similar SBP and diabetes risk (Figs. 1E and 2E). The estimates for pulse pressure and plasma apoB were significantly different by age (Fig. 1E, Supplementary Table 3) and those for HbA1c and plasma apoB were significantly different by sex (Fig. 2E, Supplementary Table 4). Results for blood pressure without adjustment for medication use were similar (Supplementary Table 9).

The interactions of sex and age in the associations of *APOE* genotypes with all outcomes were significant (Supplementary Table 10).

## Discussion

Consistent with previous studies, compared to the ε3ε3 genotype, ε2ε3 had lower risk of IHD^16,23–26^, lower SBP and lower plasma apoB^13^. ε3ε4 and ε4ε4 had higher IHD risk^16,23–26^ and plasma apoB^29–32^, but lower HbA1c or diabetes risk^14^, while ε2ε2 and ε2ε4 had similar risk of IHD as ε3ε3^13^. *APOE* genotypes also had the expected associations with LDL cholesterol^23^. This study adds by showing ε2ε3 had lower pulse pressure than ε3ε3. ε3ε4 and ε4ε4 carriers had higher pulse pressure. Finally, this study suggests ε2ε2 might be harmful for IHD in younger people. In addition, some of the associations with blood pressure and plasma apoB differed by sex.

Previous studies have found similar associations of these *APOE* genotypes with IHD^16,23–26^, including ε2ε2 having unclear effects on IHD^13,14^. Here we cannot exclude the possibility that ε2ε2 compared with ε2ε3 is harmful in younger people. ε2ε2 carriers were younger at recruitment than other genotypes carriers, which indicates poorer survival and a harmful genotype, given older cohorts would theoretically have fewer ε2ε2 carriers due to the effect of harmful phenotypes, here possibly on IHD^47^. Given the ε2ε2 genotype is associated with hyperlipoproteinemia^48,49^, peripheral vascular disease and thromboembolism^14^, the observed overall null association of ε2ε2 compared to ε2ε3 with IHD could be the result of selection bias, due to death from effects of this genotype or from other risk factors for IHD precluding recruitment, and thereby obscuring a possibly harmful effect (in the younger group). Findings on blood pressure are somewhat consistent with a previous PheWAS, although they did not include pulse pressure, did not account for effects of anti-hypertensive medications when drug use differs across genotypes (Table 1) and only considered the first five principal components^13^. The associations of ε2ε2 genotype with IHD and blood pressure in men and women were in different directions, although most of these differences were not significant. Our findings for the associations of *APOE* genotypes with diabetes and HbA1c are internally consistent and consistent with the recent PheWAS^14^, but are inconsistent with a previous meta-analysis mainly in Asians suggesting ε4 carriers had higher risk of diabetes than ε3ε3 carriers^28^, and with a cross-population meta-analysis suggesting ε2 carriers had higher risk than others^50^. Our study in an ethnically homogenous population is less open to any potential biases from population stratification. Several previous studies have also reported similar associations of *APOE* genotype with plasma apoB^29–32^.

Plasma apoB is emerging as an important cause of IHD^37–39^. At this moment, the inter-relationship between *APOE* and plasma apoB is unclear, as is the role, if any, that products of the *APOE* genotypes have in determining plasma apoB. Similarly, whether *APOE* might affect blood pressure, and the corresponding mechanism, is not well studied. The *APOE* gene has high expression in the adrenal gland^51^. Newly synthesized apoE protein has been found in the kidney and adrenal cortex^52^, which might be relevant to its effect on blood pressure. In future it would be informative to use multivariable MR to test the effect of each genotype on IHD independent of SBP, and the linearity of these associations. We also found some sex differences in associations of *APOE* genotypes with IHD risk factors, specifically blood pressure and plasma apoB. Sex hormones, such as androgens, increase plasma apoB^53^, which could be one of the pathways. Whether the complex pattern of associations of ε2ε2 with blood pressure are relevant to sex difference or merely chance findings, however, might deserve clarification. At baseline the associations with BMI, physical activity, and medication use differed somewhat by genotype, indicating a co-morbidity burden and/or secondary CVD effect of *APOE* genotypes.

Diabetes undoubtedly causes IHD, but differing directions of associations for risk factors with IHD and diabetes are surprisingly common, as found here for ε4 genotypes, suggesting that factors protecting against diabetes but also causing IHD might exist. A similar pattern has also been observed for statins^54,55^, diuretics^56,57^ and familial hypercholesteremia^58,59^, where statins and diuretics protect against IHD and impair glucose metabolism, while familial hypercholesteremia causes IHD and protects against diabetes. Statins increase LDL receptor expression^60^, and the LDL receptor has high affinity for apoE4. *LDLR* and *APOE* gene mutations also cause familial hypercholesterolemia^58,61^. Statins affect hormones^62^, how *APOE* affects hormones has not been comprehensively investigated, although possible mechanistic pathways exist^51^. *APOE* genotypes affect androgens^18^, and androgen affects plasma apoB^53^ and HbA1c^63^ in different directions, whose relevance needs further investigation.

### Strengths and limitations

This study is based on a very large population-based cohort which has the advantage of being relatively young and enabling stratification by age. We controlled for population stratification in several different ways, including restricting the analysis to white British people, excluding people with extensive relatedness and adjusting for principal components. We found little relation of *APOE* genotypes with potential confounders such as socioeconomic position, except that ε4ε4 carriers had a lower BMI and were more physically active, possibly as a consequence of changes in lifestyle indicated by their high LDL cholesterol in this study or selection bias because we inevitably excluded people who died of their ε4ε4 genotype before recruitment. This is a cross sectional study design, but genetic variants are unlikely to be affected by common confounders such as socioeconomic position, lifestyle or health status, as well as the disease outcomes or biomarkers, reducing vulnerability to confounding. However, genetic studies could be open to selection bias, here due to the inevitable recruitment of people who have survived to age 40–69 years. Notably, the ε3ε3 genotype was associated with older age at recruitment and the ε2 and ε4 homozygous genotypes with younger age at recruitment, suggesting missing older people with ε2 and ε4 homozygous genotypes, meaning the observed effects for them may be attenuated (smaller observed effects) or even reversed, depending on the magnitude of selection bias. The analysis was restricted to the participants of European ancestry, whether the associations apply to other populations are uncertain. However, mechanisms should be consistent across populations although they might be not relevant in all settings^64^. In the analyses stratified by age at recruitment, 60 years is a slightly arbitrary cut-off. Given increasing IHD prevalence by age we could not create age stratified groups with similar IHD prevalence, but we created groups with similar numbers of events in each group, which gave an age cut-off of 63 years, results were similar using this revised cut-off. Lastly, the number of people with the ε2ε2 genotype may have been inadequate to fully elucidate its role.

## Conclusions

*APOE* genotypes affect risk of IHD, with approximately lowest to highest risk as ε2ε3, ε3ε3, ε3ε4 and ε4ε4 overall, while the ε2ε2 genotype might be harmful for IHD in younger people. The association of these genotypes with major IHD risk factors, including blood pressure and diabetes however, was not always in the same pattern overall, and some associations differed by sex, highlighting the complexity of IHD etiology, as well as the importance of investigating the role of products of *APOE* sex-specifically as potential targets of intervention for cardiovascular disease and possibly diabetes prevention.

## Supplementary Information

Supplementary Information

## Data Availability

The data underlying this article are from the UK Biobank under application (42,468). The data is available from the UK Biobank upon request.

## Code availability

The R code used in this study are available from the corresponding author, upon reasonable request.

## References

[1] GBD Causes of Death Collaborators (2017). Global regional, and national age-sex specific mortality for 264 causes of death, 1980–2016: a systematic analysis for the Global Burden of Disease Study 2016. Lancet.

[2] Bloom DE (2011). The Global Economic Burden of Noncommunicable Diseases.

[3] Voight BF (2012). Plasma HDL cholesterol and risk of myocardial infarction: a mendelianrandomisation study. Lancet (London, England).

[4] Kathiresan S, Srivastava D (2012). Genetics of human cardiovascular disease. Cell.

[5] Shu L, Blencowe M, Yang X (2018). Translating GWAS findings to novel therapeutic targets for coronary artery disease. Front. Cardiovasc. Med..

[6] Swerdlow DI, Holmes MV, Harrison S, Humphries SE (2012). The genetics of coronary heart disease. Br. Med. Bull..

[7] Mozaffarian D (2016). executive summary: heart disease and stroke statistics—2016 update. Circulation.

[8] Bartz D (2020). Clinical advances in sex- and gender-informed medicine to improve the health of all: a review. JAMA Intern. Med..

[9] Sidney S (2016). Recent trends in cardiovascular mortality in the United States and public health goals. JAMA Cardiol..

[10] Lloyd-Jones DM (2016). Slowing progress in cardiovascular mortality rates: you reap what you sow. JAMA Cardiol..

[11] Li T (2019). Discontinued drugs for the treatment of cardiovascular disease from 2016 to 2018. Int. J. Mol. Sci..

[12] Reilly M, Rader DJ (2006). Apolipoprotein E and coronary disease: a puzzling paradox. PLoS Med..

[13] Kuo CL, Pilling LC, Atkins JL, Kuchel GA, Melzer D (2020). ApoE e2 and aging-related outcomes in 379,000 UK Biobank participants. Aging (Albany NY).

[14] Lumsden AL, Mulugeta A, Zhou A, Hyppönen E (2020). Apolipoprotein E (APOE) genotype-associated disease risks: a phenome-wide, registry-based, case-control study utilising the UK Biobank. EBioMedicine.

[15] Phillips MC (2014). Apolipoprotein E isoforms and lipoprotein metabolism. IUBMB Life.

[16] Zhao QR, Lei YY, Li J, Jiang N, Shi JP (2017). Association between apolipoprotein E polymorphisms and premature coronary artery disease: a meta-analysis. Clin. Chem. Lab. Med..

[17] Sebastiani P (2018). APOE alleles and extreme human longevity. J. Gerontol. Ser. A.

[18] Zofková I, Zajícková K, Hill M, Horínek A (2002). Apolipoprotein E gene determines serum testosterone and dehydroepiandrosterone levels in postmenopausal women. Eur. J. Endocrinol..

[19] Schooling CM (2017). Practical applications of evolutionary biology in public health. Lancet.

[20] Eisenberg DT, Kuzawa CW, Hayes MG (2010). Worldwide allele frequencies of the human apolipoprotein E gene: climate, local adaptations, and evolutionary history. Am. J. Phys. Anthropol..

[21] Finch CE, Sapolsky RM (1999). The evolution of Alzheimer disease, the reproductive schedule, and apoE isoforms. Neurobiol. Aging.

[22] Mahley RW, Rall SC (1999). Is epsilon4 the ancestral human apoE allele?. Neurobiol. Aging.

[23] Bennet AM (2007). Association of apolipoprotein e genotypes with lipid levels and coronary risk. JAMA.

[24] Zhang Y, Tang H-Q, Peng W-J, Zhang B-B, Liu M (2015). Meta-analysis for the association of apolipoprotein E ε2/ε3/ε4 polymorphism with coronary heart disease. Chin. Med. J. (Engl.).

[25] Xu H (2014). Meta-analysis of apolipoprotein E gene polymorphism and susceptibility of myocardial infarction. PLoS ONE.

[26] Xu M (2016). Apolipoprotein E gene variants and risk of coronary heart disease: a meta-analysis. Biomed. Res. Int..

[27] Shi J (2018). Association between ApoE polymorphism and hypertension: a meta-analysis of 28 studies including 5898 cases and 7518 controls. Gene.

[28] Chen DW, Shi JK, Li Y, Yang Y, Ren SP (2019). Association between ApoE polymorphism and type 2 diabetes: a meta-analysis of 59 studies. Biomed. Environ. Sci..

[29] Boerwinkle E, Utermann G (1988). Simultaneous effects of the apolipoprotein E polymorphism on apolipoprotein E, apolipoprotein B, and cholesterol metabolism. Am. J. Hum. Genet..

[30] Griffin BA (2018). APOE4 genotype exerts greater benefit in lowering plasma cholesterol and apolipoprotein B than wild type (E3/E3), after replacement of dietary saturated fats with low glycaemic index carbohydrates. Nutrients.

[31] Khan TA (2013). Apolipoprotein E genotype, cardiovascular biomarkers and risk of stroke: systematic review and meta-analysis of 14,015 stroke cases and pooled analysis of primary biomarker data from up to 60,883 individuals. Int. J. Epidemiol..

[32] Soares HD (2012). Plasma biomarkers associated with the apolipoprotein E genotype and Alzheimer disease. Arch. Neurol..

[33] Wilson PW, Schaefer EJ, Larson MG, Ordovas JM (1996). Apolipoprotein E alleles and risk of coronary disease. A meta-analysis. ArteriosclerThromb. Vasc. Biol..

[34] Licastro F (2004). The concomitant presence of polymorphic alleles of interleukin-1beta, interleukin-6 and apolipoprotein E is associated with an increased risk of myocardial infarction in elderly men. Results from a pilot study. Mech. Ageing Dev..

[35] Heijmans BT (2002). Association of APOE epsilon2/epsilon3/epsilon4 and promoter gene variants with dementia but not cardiovascular mortality in old age. Am. J. Med. Genet..

[36] Zhao JV, Schooling CM (2018). Coagulation factors and the risk of ischemic heart disease: a mendelian randomization study. Circ. GenomPrecis. Med..

[37] Ference BA (2019). Association of triglyceride-lowering LPL variants and LDL-C-lowering LDLR variants with risk of coronary heart disease. JAMA.

[38] Richardson TG (2020). Evaluating the relationship between circulating lipoprotein lipids and apolipoproteins with risk of coronary heart disease: a multivariable Mendelian randomisation analysis. PLoS Med..

[39] Ference BA (2017). Association of genetic variants related to CETP inhibitors and statins with lipoprotein levels and cardiovascular risk. JAMA.

[40] Sudlow C (2015). UK biobank: an open access resource for identifying the causes of a wide range of complex diseases of middle and old age. PLoS Med..

[41] Bycroft C (2018). The UK Biobank resource with deep phenotyping and genomic data. Nature.

[42] Evangelou E (2018). Genetic analysis of over 1 million people identifies 535 new loci associated with blood pressure traits. Nat. Genet..

[43] Baigent C (2005). Efficacy and safety of cholesterol-lowering treatment: prospective meta-analysis of data from 90,056 participants in 14 randomised trials of statins. Lancet.

[44] UK Biobank GWAS results, http://www.nealelab.is/uk-biobank/ (2018).

[45] Ageing and health, https://www.who.int/news-room/fact-sheets/detail/ageing-and-health (2018).

[46] Paternoster R, Brame R, Mazerolle P, Piquero A (1998). Using the correct statistical test for the equality of regression coefficients. Criminology.

[47] Heijmans BT, Westendorp RG, Slagboom PE (2000). Common gene variants, mortality and extreme longevity in humans. Exp. Gerontol..

[48] Zannis VI (1986). Genetic polymorphism in human apolipoprotein E. Methods Enzymol..

[49] Koopal C, Marais AD, Visseren FLJ (2017). Familial dysbetalipoproteinemia: an underdiagnosed lipid disorder. Curr. Opin. Endocrinol. Diabetes Obes..

[50] Anthopoulos PG, Hamodrakas SJ, Bagos PG (2010). Apolipoprotein E polymorphisms and type 2 diabetes: a meta-analysis of 30 studies including 5423 cases and 8197 controls. Mol. Genet. Metab..

[51] Yao C (2018). Genome-wide mapping of plasma protein QTLs identifies putatively causal genes and pathways for cardiovascular disease. Nat. Commun..

[52] Blue ML, Williams DL, Zucker S, Khan SA, Blum CB (1983). Apolipoprotein E synthesis in human kidney, adrenal gland, and liver. Proc. Natl. Acad. Sci. U.S.A..

[53] Hartgens F, Rietjens G, Keizer HA, Kuipers H, Wolffenbuttel BH (2004). Effects of androgenic-anabolic steroids on apolipoproteins and lipoprotein (a). Br. J. Sports Med..

[54] Vallejo-Vaz AJ (2017). Low-density lipoprotein cholesterol lowering for the primary prevention of cardiovascular disease among men with primary elevations of low-density lipoprotein cholesterol levels of 190 mg/dL or above: analyses from the WOSCOPS (west of scotland coronary prevention study) 5-year randomized trial and 20-year observational follow-up. Circulation.

[55] Ridker PM, Pradhan A, MacFadyen JG, Libby P, Glynn RJ (2012). Cardiovascular benefits and diabetes risks of statin therapy in primary prevention: an analysis from the JUPITER trial. Lancet.

[56] Pitt B (1999). The effect of spironolactone on morbidity and mortality in patients with severe heart failure. Randomized aldactone evaluation study investigators. N. Engl. J. Med..

[57] Elliott WJ, Meyer PM (2007). Incident diabetes in clinical trials of antihypertensive drugs: a network meta-analysis. Lancet.

[58] Nordestgaard BG (2013). Familial hypercholesterolaemia is underdiagnosed and undertreated in the general population: guidance for clinicians to prevent coronary heart disease: consensus statement of the European Atherosclerosis Society. Eur. Heart J..

[59] Besseling J, Kastelein JJ, Defesche JC, Hutten BA, Hovingh GK (2015). Association between familial hypercholesterolemia and prevalence of type 2 diabetes mellitus. JAMA.

[60] Preiss D, Sattar N (2015). Does the LDL receptor play a role in the risk of developing type 2 diabetes?. JAMA.

[61] Ghaleb Y (2018). Usefulness of the genetic risk score to identify phenocopies in families with familial hypercholesterolemia?. Eur. J. Hum. Genet..

[62] Schooling CM, Au Yeung SL, Freeman G, Cowling BJ (2013). The effect of statins on testosterone in men and women, a systematic review and meta-analysis of randomized controlled trials. BMC Med..

[63] Groti K, Žuran I, Antonič B, Foršnarič L, Pfeifer M (2018). The impact of testosterone replacement therapy on glycemic control, vascular function, and components of the metabolic syndrome in obese hypogonadal men with type 2 diabetes. Aging Male.

[64] Rothman KJ, Gallacher JE, Hatch EE (2013). Why representativeness should be avoided. Int. J. Epidemiol..

