# Supplementary material for: Age and sex specific effects of APOE genotypes on ischemic heart disease and its risk factors in the UK Biobank
Source: Sci Rep. 2021 Apr 29;11:9229. doi: 10.1038/s41598-021-88256-x (PMC8085204; doi:10.1038/s41598-021-88256-x)
Supplement: Supplementary file 1 — Supplementary Information [file 41598_2021_88256_MOESM1_ESM.docx]

Age and sex specific effects of *APOE* genotypes on ischemic heart disease and its risk factors in the UK Biobank

Mengyu Li^1^, MB; Jie V Zhao^1^, PhD; Maggie Man Ki Kwok^1^, PhD; C Mary Schooling^1,2,^ *, PhD.

1 School of Public Health, Li Ka Shing Faculty of Medicine, The University of Hong Kong, Hong Kong, China

2 Graduate School of Public Health and Health Policy, City University of New York, New York, NY 10017, USA

*Corresponding Author: C Mary Schooling, PhD.

Mailing address: 1/F, Patrick Manson Building (North Wing), 7 Sassoon Road, Pokfulam, Hong Kong

Tel: 852 3917 6732

Fax: 852 3917 6732

Email: [cms1@hku.hk](mailto:cms1@hku.hk)

| Supplementary table 1 Definition of the common APOE genotypes by two single-nucleotide polymorphisms. | | |
| --- | --- | --- |
| APOE genotype | rs429358 | rs7412 |
| ε2ε2 | (T;T) | (T;T) |
| ε2ε3 | (T;T) | (C;T) |
| ε2ε4 | (C;T) | (C;T) |
| ε3ε3 | (T;T) | (C;C) |
| ε3ε4 | (C;T) | (C;C) |
| ε4ε4 | (C;C) | (C;C) |

Supplementary table 2 Algorithms for disease outcomes in the UK Biobank

| Outcome | ICD-9 codes | ICD-10 codes | Self-report codes |
| --- | --- | --- | --- |
| Ischemic heart disease | 410-414 | I20-25 | 1074, 1075 |
| Type 2 diabetes | 250 | E11-E11.9 | 1223 |

Supplementary table 3 Comparisons of differences in estimates of *APOE* genotypes by age group.

| Genotypes | Outcome | Estimate.young | SE.young | Estimate.old | SE.old | Difference | z | P_d |
| --- | --- | --- | --- | --- | --- | --- | --- | --- |
| ε2ε2 | IHD | 0.22926 | 0.119398 | -0.12737 | 0.094963 | 0.356631 | 2.337683 | 0.019404 |
| ε2ε2 | SBP | -0.64121 | 0.495715 | -0.36255 | 0.636852 | -0.27865 | -0.34528 | 0.729887 |
| ε2ε2 | DBP | 0.016792 | 0.305435 | 0.061365 | 0.345248 | -0.04457 | -0.0967 | 0.922968 |
| ε2ε2 | PP | -0.65793 | 0.309306 | -0.44113 | 0.451263 | -0.2168 | -0.39628 | 0.6919 |
| ε2ε2 | T2DM | -0.03813 | 0.158383 | -0.16896 | 0.129995 | 0.130831 | 0.638512 | 0.52314 |
| ε2ε2 | hba1c | -0.15492 | 0.165544 | -0.11351 | 0.205799 | -0.0414 | -0.15676 | 0.875431 |
| ε2ε2 | apob | -0.42191 | 0.006381 | -0.41612 | 0.007545 | -0.00578 | -0.58518 | 0.558426 |
| ε2ε2 | LDL | -0.93455 | 0.02081 | -0.88556 | 0.023131 | -0.04899 | -1.57461 | 0.115347 |
| ε2ε3 | IHD | -0.13419 | 0.035095 | -0.17631 | 0.023282 | 0.042113 | 0.99993 | 0.317344 |
| ε2ε3 | SBP | -0.40871 | 0.124747 | -0.43665 | 0.15681 | 0.027947 | 0.139473 | 0.889076 |
| ε2ε3 | DBP | -0.0926 | 0.076863 | 0.043311 | 0.08505 | -0.13591 | -1.1856 | 0.235779 |
| ε2ε3 | PP | -0.316 | 0.077837 | -0.47983 | 0.111113 | 0.163824 | 1.207571 | 0.227212 |
| ε2ε3 | T2DM | -0.0308 | 0.040126 | -0.01674 | 0.029751 | -0.01406 | -0.28153 | 0.778303 |
| ε2ε3 | hba1c | -0.06381 | 0.042057 | -0.0943 | 0.050549 | 0.030491 | 0.463685 | 0.642873 |
| ε2ε3 | apob | -0.1367 | 0.001529 | -0.1186 | 0.001768 | -0.0181 | -7.74546 | 9.52E-15 |
| ε2ε3 | LDL | -0.43468 | 0.005299 | -0.40178 | 0.005646 | -0.03289 | -4.24805 | 2.16E-05 |
| ε2ε4 | IHD | -0.0426 | 0.068758 | -0.08254 | 0.046968 | 0.039932 | 0.479558 | 0.631542 |
| ε2ε4 | SBP | -0.58628 | 0.251359 | -0.60167 | 0.323592 | 0.015392 | 0.037565 | 0.970034 |
| ε2ε4 | DBP | -0.30304 | 0.154875 | -0.2612 | 0.175509 | -0.04184 | -0.17876 | 0.85813 |
| ε2ε4 | PP | -0.28313 | 0.156837 | -0.34038 | 0.229292 | 0.057243 | 0.206059 | 0.836744 |
| ε2ε4 | T2DM | 0.057837 | 0.078204 | 0.005964 | 0.061044 | 0.051873 | 0.522872 | 0.601064 |
| ε2ε4 | hba1c | -0.07417 | 0.084819 | -0.00176 | 0.104234 | -0.07241 | -0.5388 | 0.590026 |
| ε2ε4 | apob | -0.07258 | 0.003082 | -0.06742 | 0.003658 | -0.00516 | -1.07799 | 0.28104 |
| ε2ε4 | LDL | -0.23886 | 0.010676 | -0.22823 | 0.011695 | -0.01062 | -0.67087 | 0.502303 |
| ε3ε4 | IHD | 0.108615 | 0.025011 | 0.0852 | 0.016946 | 0.023415 | 0.77505 | 0.43831 |
| ε3ε4 | SBP | 0.11534 | 0.096499 | 0.09261 | 0.122758 | 0.022731 | 0.145574 | 0.884257 |
| ε3ε4 | DBP | -0.0342 | 0.059457 | -0.23451 | 0.06658 | 0.200311 | 2.244019 | 0.024831 |
| ε3ε4 | PP | 0.149195 | 0.060212 | 0.326572 | 0.086984 | -0.17738 | -1.67668 | 0.093605 |
| ε3ε4 | T2DM | -0.10041 | 0.031696 | -0.0502 | 0.023507 | -0.05021 | -1.27238 | 0.203239 |
| ε3ε4 | hba1c | -0.16487 | 0.032529 | -0.15157 | 0.039535 | -0.0133 | -0.25977 | 0.795039 |
| ε3ε4 | apob | 0.055376 | 0.00118 | 0.044789 | 0.001382 | 0.010588 | 5.826634 | 5.66E-09 |
| ε3ε4 | LDL | 0.176127 | 0.004093 | 0.157115 | 0.004416 | 0.019012 | 3.157579 | 0.001591 |
| ε4ε4 | IHD | 0.11233 | 0.066413 | 0.162562 | 0.044664 | -0.05023 | -0.62762 | 0.530255 |
| ε4ε4 | SBP | 0.061407 | 0.260408 | 0.738532 | 0.334281 | -0.67712 | -1.59797 | 0.11005 |
| ε4ε4 | DBP | -0.26617 | 0.160434 | -0.24769 | 0.181306 | -0.01848 | -0.07634 | 0.939152 |
| ε4ε4 | PP | 0.323289 | 0.162484 | 0.986284 | 0.236866 | -0.66299 | -2.30816 | 0.02099 |
| ε4ε4 | T2DM | -0.18569 | 0.089818 | -0.11494 | 0.066035 | -0.07075 | -0.63467 | 0.525644 |
| ε4ε4 | hba1c | -0.18327 | 0.087673 | -0.36886 | 0.107667 | 0.185592 | 1.336651 | 0.181337 |
| ε4ε4 | apob | 0.096737 | 0.003166 | 0.075853 | 0.003758 | 0.020884 | 4.250371 | 2.13E-05 |
| ε4ε4 | LDL | 0.298711 | 0.010961 | 0.261664 | 0.011998 | 0.037047 | 2.279681 | 0.022627 |

Supplementary table 4 Comparisons of differences in estimates of *APOE* genotypes by sex.

| Genotypes | Outcome | Estimate.men | SE.men | Estimate.women | SE.women | Difference | z | P_d |
| --- | --- | --- | --- | --- | --- | --- | --- | --- |
| ε2ε2 | IHD | 0.03259 | 0.09305 | -0.05982 | 0.12597 | 0.09241 | 0.59009 | 0.55513 |
| ε2ε2 | SBP | -1.43599 | 0.57409 | 0.22894 | 0.54533 | -1.66493 | -2.10268 | 0.03549 |
| ε2ε2 | DBP | -0.31260 | 0.34171 | 0.32046 | 0.30813 | -0.63305 | -1.37585 | 0.16887 |
| ε2ε2 | PP | -1.12345 | 0.38117 | -0.10562 | 0.36786 | -1.01783 | -1.92141 | 0.05468 |
| ε2ε2 | T2DM | -0.13391 | 0.13127 | -0.10007 | 0.15646 | -0.03384 | -0.16571 | 0.86839 |
| ε2ε2 | hba1c | -0.13099 | 0.21962 | -0.13998 | 0.15193 | 0.00899 | 0.03368 | 0.97313 |
| ε2ε2 | apob | -0.41818 | 0.00742 | -0.42041 | 0.00648 | 0.00223 | 0.22637 | 0.82092 |
| ε2ε2 | LDL | -0.85460 | 0.02292 | -0.96042 | 0.02096 | 0.10582 | 3.40694 | 0.00066 |
| ε2ε3 | IHD | -0.18574 | 0.02408 | -0.12236 | 0.03268 | -0.06338 | -1.56138 | 0.11843 |
| ε2ε3 | SBP | -0.49172 | 0.14129 | -0.36296 | 0.13730 | -0.12876 | -0.65358 | 0.51338 |
| ε2ε3 | DBP | -0.03398 | 0.08410 | -0.02539 | 0.07761 | -0.00859 | -0.07505 | 0.94017 |
| ε2ε3 | PP | -0.45784 | 0.09381 | -0.33725 | 0.09262 | -0.12059 | -0.91477 | 0.36031 |
| ε2ε3 | T2DM | -0.01903 | 0.03045 | -0.02810 | 0.03857 | 0.00907 | 0.18447 | 0.85364 |
| ε2ε3 | hba1c | -0.09090 | 0.05438 | -0.06651 | 0.03836 | -0.02439 | -0.36654 | 0.71396 |
| ε2ε3 | apob | -0.11676 | 0.00174 | -0.13831 | 0.00155 | 0.02155 | 9.24619 | 2.33E-20 |
| ε2ε3 | LDL | -0.36957 | 0.00564 | -0.46232 | 0.00530 | 0.09276 | 11.97841 | 4.61E-33 |
| ε2ε4 | IHD | -0.05756 | 0.04763 | -0.09445 | 0.06700 | 0.03689 | 0.44881 | 0.65357 |
| ε2ε4 | SBP | -0.74083 | 0.28771 | -0.47172 | 0.27969 | -0.26911 | -0.67067 | 0.50243 |
| ε2ε4 | DBP | -0.33058 | 0.17125 | -0.24067 | 0.15810 | -0.08991 | -0.38576 | 0.69968 |
| ε2ε4 | PP | -0.41033 | 0.19103 | -0.23076 | 0.18867 | -0.17957 | -0.66882 | 0.50361 |
| ε2ε4 | T2DM | 0.02637 | 0.06138 | 0.02618 | 0.07763 | 0.00019 | 0.00195 | 0.99845 |
| ε2ε4 | hba1c | -0.03318 | 0.11044 | -0.04793 | 0.07834 | 0.01474 | 0.10889 | 0.91329 |
| ε2ε4 | apob | -0.06781 | 0.00353 | -0.07233 | 0.00318 | 0.00452 | 0.95163 | 0.34129 |
| ε2ε4 | LDL | -0.20857 | 0.01147 | -0.25610 | 0.01085 | 0.04754 | 3.01051 | 0.00261 |
| ε3ε4 | IHD | 0.11397 | 0.01733 | 0.05230 | 0.02393 | 0.06167 | 2.08674 | 0.03691 |
| ε3ε4 | SBP | -0.07386 | 0.10984 | 0.25698 | 0.10682 | -0.33084 | -2.15926 | 0.03083 |
| ε3ε4 | DBP | -0.23941 | 0.06538 | -0.02566 | 0.06038 | -0.21375 | -2.40179 | 0.01632 |
| ε3ε4 | PP | 0.16493 | 0.07293 | 0.28238 | 0.07206 | -0.11745 | -1.14557 | 0.25197 |
| ε3ε4 | T2DM | -0.07080 | 0.02413 | -0.06272 | 0.03031 | -0.00809 | -0.20876 | 0.83464 |
| ε3ε4 | hba1c | -0.18730 | 0.04228 | -0.13442 | 0.02981 | -0.05288 | -1.02212 | 0.30673 |
| ε3ε4 | apob | 0.04479 | 0.00135 | 0.05562 | 0.00121 | -0.01083 | -5.99062 | 2.09E-09 |
| ε3ε4 | LDL | 0.13951 | 0.00438 | 0.19161 | 0.00412 | -0.05210 | -8.66172 | 4.65E-18 |
| ε4ε4 | IHD | 0.18451 | 0.04516 | 0.07493 | 0.06503 | 0.10958 | 1.38414 | 0.16632 |
| ε4ε4 | SBP | 0.25091 | 0.29640 | 0.44140 | 0.29046 | -0.19050 | -0.45904 | 0.64620 |
| ε4ε4 | DBP | -0.42332 | 0.17640 | -0.12375 | 0.16418 | -0.29957 | -1.24312 | 0.21382 |
| ε4ε4 | PP | 0.66902 | 0.19679 | 0.56543 | 0.19593 | 0.10359 | 0.37304 | 0.70912 |
| ε4ε4 | T2DM | -0.10818 | 0.06639 | -0.19365 | 0.08901 | 0.08546 | 0.76965 | 0.44151 |
| ε4ε4 | hba1c | -0.52636 | 0.11438 | -0.04521 | 0.08079 | -0.48115 | -3.43595 | 0.00059 |
| ε4ε4 | apob | 0.07612 | 0.00362 | 0.09728 | 0.00327 | -0.02117 | -4.33854 | 0.00001 |
| ε4ε4 | LDL | 0.22980 | 0.01176 | 0.32825 | 0.01115 | -0.09845 | -6.07634 | 1.23E-09 |

Supplementary table 5 Effects of *APOE* ε2ε2 genotype compared with ε3ε3 on blood pressure and low-density lipoprotein cholesterol without adjustment for medication use.

| Outcome | Participants | Beta | 95% CI | P |
| --- | --- | --- | --- | --- |
| SBP | all | -0.40 | -1.11, 0.31 | 0.27 |
|  | men | -1.25 | -2.28, -0.22 | 0.02 |
|  | women | 0.30 | -0.69, 1.28 | 0.56 |
|  | younger | -0.64 | -1.54, 0.26 | 0.16 |
|  | older | -0.09 | -1.23, 1.04 | 0.87 |
| DBP | all | 0.12 | -0.29, 0.52 | 0.58 |
|  | men | -0.19 | -0.80, 0.42 | 0.54 |
|  | women | 0.36 | -0.19, 0.90 | 0.20 |
|  | younger | 0.02 | -0.53, 0.57 | 0.95 |
|  | older | 0.23 | -0.37, 0.84 | 0.45 |
| PP | all | -0.53 | -1.03, -0.02 | 0.04 |
|  | men | -1.06 | -1.79, -0.33 | 0.00 |
|  | women | -0.08 | -0.79, 0.62 | 0.82 |
|  | younger | -0.66 | -1.25, -0.06 | 0.03 |
|  | older | -0.35 | -1.21, 0.51 | 0.42 |
| LDL | all | -0.90 | -0.93, -0.86 | 0 |
|  | men | -0.84 | -0.89, -0.79 | 8.76E-233 |
|  | women | -0.94 | -0.99, -0.90 | 0 |
|  | younger | -0.94 | -0.99, -0.90 | 0 |
|  | older | -0.84 | -0.89, -0.78 | 8.21E-207 |

Supplementary table 6 Effects of *APOE* ε2ε3 genotype compared with ε3ε3 on blood pressure and low-density lipoprotein cholesterol without adjustment for medication use.

| Outcome | Participants | Beta | 95% CI | P |
| --- | --- | --- | --- | --- |
| SBP | all | -0.27 | -0.45, -0.10 | 2.60E-03 |
|  | men | -0.30 | -0.56, -0.05 | 0.02 |
|  | women | -0.25 | -0.50, 0.00 | 0.05 |
|  | younger | -0.30 | -0.53, -0.07 | 0.01 |
|  | older | -0.24 | -0.52, 0.04 | 0.09 |
| DBP | all | 0.07 | -0.03, 0.17 | 0.17 |
|  | men | 0.09 | -0.06, 0.24 | 0.22 |
|  | women | 0.05 | -0.09, 0.19 | 0.46 |
|  | younger | -0.02 | -0.16, 0.12 | 0.79 |
|  | older | 0.17 | 0.03, 0.32 | 0.02 |
| PP | all | -0.34 | -0.47, -0.22 | 1.04E-07 |
|  | men | -0.39 | -0.57, -0.22 | 1.61E-05 |
|  | women | -0.30 | -0.48, -0.12 | 9.43E-04 |
|  | younger | -0.28 | -0.43, -0.13 | 2.45E-04 |
|  | older | -0.41 | -0.63, -0.20 | 1.27E-04 |
| LDL | all | -0.37 | -0.38, -0.36 | 0 |
|  | men | -0.31 | -0.33, -0.30 | 0 |
|  | women | -0.42 | -0.43, -0.41 | 0 |
|  | younger | -0.41 | -0.42, -0.40 | 0 |
|  | older | -0.32 | -0.34, -0.31 | 0 |

Supplementary table 7 Effects of *APOE* ε2ε4 genotype compared with ε3ε3 on blood pressure and low-density lipoprotein cholesterol without adjustment for medication use.

| Outcome | Participants | Beta | 95% CI | P |
| --- | --- | --- | --- | --- |
| SBP | all | -0.46 | -0.83, -0.10 | 0.01 |
|  | men | -0.68 | -1.20, -0.16 | 0.01 |
|  | women | -0.28 | -0.78, 0.22 | 0.28 |
|  | younger | -0.54 | -0.99, -0.08 | 0.02 |
|  | older | -0.37 | -0.95, 0.21 | 0.21 |
| DBP | all | -0.19 | -0.40, 0.01 | 0.07 |
|  | men | -0.29 | -0.59, 0.02 | 0.06 |
|  | women | -0.11 | -0.39, 0.17 | 0.43 |
|  | younger | -0.27 | -0.55, 0.01 | 0.06 |
|  | older | -0.11 | -0.42, 0.20 | 0.49 |
| PP | all | -0.27 | -0.53, -0.01 | 0.04 |
|  | men | -0.39 | -0.75, -0.02 | 0.04 |
|  | women | -0.17 | -0.53, 0.19 | 0.36 |
|  | younger | -0.27 | -0.57, 0.03 | 0.08 |
|  | older | -0.26 | -0.70, 0.17 | 0.24 |
| LDL | all | -0.21 | -0.23, -0.20 | 1.01E-133 |
|  | men | -0.20 | -0.22, -0.17 | 6.03E-52 |
|  | women | -0.23 | -0.25, -0.21 | 3.66E-85 |
|  | younger | -0.23 | -0.25, -0.21 | 4.63E-94 |
|  | older | -0.20 | -0.22, -0.17 | 2.97E-46 |

Supplementary table 8 Effects of *APOE* ε3ε4 genotype compared with ε3ε3 on blood pressure and low-density lipoprotein cholesterol without adjustment for medication use.

| Outcome | Participants | Beta | 95% CI | P |
| --- | --- | --- | --- | --- |
| SBP | all | 0.08 | -0.05, 0.22 | 0.23 |
|  | men | -0.11 | -0.31, 0.09 | 0.28 |
|  | women | 0.25 | 0.06, 0.44 | 0.01 |
|  | younger | 0.06 | -0.11, 0.24 | 0.48 |
|  | older | 0.11 | -0.11, 0.33 | 0.33 |
| DBP | all | -0.14 | -0.22, -0.06 | 0.000606 |
|  | men | -0.26 | -0.38, -0.15 | 9.92E-06 |
|  | women | -0.03 | -0.14, 0.07 | 0.56 |
|  | younger | -0.07 | -0.18, 0.04 | 0.20 |
|  | older | -0.22 | -0.34, -0.11 | 0.000167 |
| PP | all | 0.22 | 0.12, 0.32 | 1.03E-05 |
|  | men | 0.15 | 0.01, 0.29 | 0.03 |
|  | women | 0.28 | 0.14, 0.42 | 7.10E-05 |
|  | younger | 0.13 | 0.02, 0.25 | 0.03 |
|  | older | 0.33 | 0.17, 0.50 | 8.74E-05 |
| LDL | all | 0.14 | 0.13, 0.14 | 0 |
|  | men | 0.10 | 0.09, 0.11 | 2.14E-99 |
|  | women | 0.16 | 0.16, 0.17 | 6.48E-295 |
|  | younger | 0.16 | 0.15, 0.16 | 2.84E-289 |
|  | older | 0.11 | 0.10, 0.12 | 8.43E-107 |

Supplementary table 9 Effects of *APOE* ε4ε4 genotype compared with ε3ε3 on blood pressure and low-density lipoprotein cholesterol without adjustment for medication use.

| Outcome | Participants | Beta | 95% CI | P |
| --- | --- | --- | --- | --- |
| SBP | all | 0.31 | -0.06, 0.69 | 0.10 |
|  | men | 0.19 | -0.35, 0.72 | 0.49 |
|  | women | 0.42 | -0.10, 0.94 | 0.12 |
|  | younger | 0.14 | -0.34, 0.61 | 0.57 |
|  | older | 0.55 | -0.05, 1.14 | 0.07 |
| DBP | all | -0.29 | -0.50, -0.07 | 0.01 |
|  | men | -0.46 | -0.78, -0.15 | 0.00 |
|  | women | -0.14 | -0.43, 0.15 | 0.35 |
|  | younger | -0.22 | -0.50, 0.07 | 0.14 |
|  | older | -0.38 | -0.69, -0.06 | 0.02 |
| PP | all | 0.60 | 0.34, 0.87 | 9.49E-06 |
|  | men | 0.65 | 0.27, 1.02 | 0.00073 |
|  | women | 0.56 | 0.18, 0.93 | 0.003507 |
|  | younger | 0.35 | 0.04, 0.66 | 0.03 |
|  | older | 0.92 | 0.47, 1.37 | 6.28E-05 |
| LDL | all | 0.23 | 0.21, 0.24 | 7.20E-140 |
|  | men | 0.17 | 0.14, 0.19 | 8.04E-36 |
|  | women | 0.28 | 0.25, 0.30 | 1.87E-115 |
|  | younger | 0.26 | 0.24, 0.28 | 4.61E-115 |
|  | older | 0.18 | 0.15, 0.21 | 5.72E-37 |

Supplementary table 10 Interaction of age and sex in associations of *APOE* genotypes on different outcomes overall.

| Outcome | Beta* | P* |
| --- | --- | --- |
| IHD | 0.099057 | 0.000809 |
| SBP | -0.13605 | 0.234331 |
| DBP | 0.394406 | 2.53E-09 |
| PP | -0.53049 | 4.48E-12 |
| T2DM | 0.126282 | 0.000207 |
| hba1c | -0.16327 | 1.45E-05 |
| apob | -0.01767 | 1.55E-39 |
| LDL | -0.08904 | 8.82E-88 |

*Beta and P values were derived from the interaction term (age and sex) in the regressions.
